# Round the Hospitals

**Published:** 1918-12-28

**Authors:** 


### ROUND THE HOSPITALS.

THE Secretary of State for War has announced the award of the Royal Red Cross, First Class, to Miss Jessie Durie, matron, and Miss Rachel Nightingale Fogarty, matron; and of the Royal Red Cross, Second Class, to Miss Eliza Covey, matron, and Miss Agnes O'Brien, sister, all of the South African Medical Corps.

THE Edmonton Board of Guardians have decided that nurses at the hospital and infirmary who may have contracted influenza or pneumonia during the recent epidemic shall be sent to the seaside to recuperate. This arrangement is subject to the recommendation of the Medical Superintendent, that the nurses concerned would benefit by the change. The Guardians are to pay full salary less any amounts received as benefit under the National Insurance Act.

THE Queen's Needlework Fund, which has been the means of producing an almost incredible wealth of comforts for the Army and Navy during the war, is to close at the end of January, and workers are asked to finish all work still on hand by the

**ROUND THE HOSPITALS**—(*continued*).

middle of the month. The orthopædic section will be kept going a little longer, since the need is likely to be felt for some time in that direction. Many other depôts are now closing down, and are disposing of surplus stocks of wool, etc., below cost prices to any who will undertake to use them for the soldiers' benefit.

The Town Council at Vienna have taken a very popular step in making use of the Imperial Castle at Schoenbrunn for the purposes of a Children's Hospital. Other of the Crown estates are to be utilised for Children's Welfare schemes. The drawback to the proposal is the difficulty of warming great corridors and suites of rooms. But the process of adapting castles for the use of sick children is one to draw out the best skill and enthusiasm the town can furnish, and when spring is at hand it may be hoped the grim old walls will enclose the happiest family which has ever found shelter within their circuit.

The "Brave Poor Things" had a delightful Christmas party on December 19th in the Chapter House of Southwark Cathedral. They consist of blind, crippled, and raid-shock children from St. Nicholas Home, Chailey, and the Hermitage Craft Schools. Many nurses from Guy's Hospital were present. An Armistice Fund is being raised to extend the work at Chailey, which is conducted on very good lines with a view to making the children as far as possible self-supporting in later years. But the name is detestable, and we cannot believe any great development of interest will accrue until it is altered. The first principle in all such work is to banish any impression among the patients that they are marked out as objects of pity.

The King held a brilliant naval and military Investiture in the Ball-room of Buckingham Palace on December 19, at 10.30. The band of the Irish Guards played during the proceedings. Officers from the Serbian, Greek, Portuguese and French Armies were present, and about five hundred relatives and friends of the recipients. Three Victoria Crosses were among the honours. The Royal Red Cross, First Class, was bestowed by the King on: Sister Ethel Harwood and Sister Mary Thomson, Q.A.I.M.N.S.R. His Majesty admitted the following to be Associates of the Order: Sisters Fanny Boulton, Mabel Campbell, Frances Hobbs, Sidney Rea, Janet Rodger, Priscilla Walker, and Staff Nurse Kate Rossi, all of Q.A.I.M.N.S.R.; Sister Fanny Ashby, T.F.N.S.; Sister Mary Quitall, C.N.S.; Miss Victoria Dunn and Miss Helena Nisbett, V.A.D. After the investiture the members of the Military Nursing Services who had been awarded the Royal Red Cross were received at Marlborough House by Queen Alexandra.

The Christmas leave of twelve days which the authorities are granting to all military patients fit to dispense with medical and nursing treatment for this short period will largely deplete the military and auxiliary hospitals next week and will, no doubt, make it easier to provide the rest with good things. The gracious relaxations of the Food Controller chime in happily at a time when Christmas festivities no longer have to be tackled against the grain with sore hearts. And on all sides there is evidence that in civil and military establishments the day will be celebrated in real old-fashioned style, with enough leaven of the new modern style to lend a zest. For very many nurses and V.A.D.s it will be their last Christmas in hospital, and memorable on that account. To many matrons and commandants it marks the passing of the hospital to which they have devoted their entire energies ever since it came into being. Already many testimonials and parting gifts are in the air. Friends bound together by the close ties of anxieties shared in common, in a life so intimate that it reveals the very bones of the soul, will soon be sundered. The years of war are already a memory, standing out clear-cut from the rest of life. The voyages gather on the brink of a new epoch, and before the vessel comes in which is to bear them to new scenes they look back and survey the chequered path which they trod together. Then, with a clasp of the hand, with eyes shining and hearts stirred, they separate. North, south, east, and west the call sounds. These who hearkened to the stern summoning of their country in war are not deaf to her trumpet-call to action in days of peace.

That the Church of England has lamentably failed of recent years to bear witness to the existence of social evils or to attack with energy the forces of wrong is the confession of the final Committee of Inquiry appointed by the Archbishop in connection with the National Mission. The chairman was Dr. Talbot, Bishop of Winchester, and the report of the Committee dealing with industrial questions, political economy, and education has recently been issued, and can be obtained from the S.P.C.K. at the price of 1s. Two women, Miss Irene Cox and Miss Constance Smith, were members of the Committee, which was by no means exclusively clerical, and it contains much which Churchmen and Churchwomen would do well to ponder over. The principle laid down that "industry ought to be regarded primarily as a social service, based on the effort of every individual to discharge his duty to his neighbour and to the community," has a wide application to the nursing profession, and cuts at the root of that narrow theory which would make self-interest the moving spring of the nurse's career. Side by side with this comes the principle that the living wage implies sufficient to maintain the worker "in health and honour, with such a margin of leisure as will permit reasonable recreation and the development of mind and spirit." The carrying out of this principle in the nursing profession is the thing to fight for.

The *Times* brings fresh statistics of the death-roll from influenza all over the world, which is now estimated at 6,000,000 during the last twelve

**ROUND THE HOSPITALS—(*continued*).**

weeks. The deaths in London alone from this cause were over 10,000, while the total loss of life in the air raids amounted to a few hundred only. In asserting that "never since the Black Death has such a plague swept over the face of the earth," the writer perhaps omits to consider the fact that only during this generation has the preparation of vital statistics been a world-wide care. But the reports collected from the Antipodes and the East show that the ravages of influenza have not been exaggerated. In India alone there have been over 3,000,000 deaths. In Cape Town 2,000 children have been left destitute by the death of their parents, "while the plague swept through the native areas like fire." In Samoa 80 per cent. of the natives were affected. In Canada the disease assumed a very virulent form, and in Ontario and the Western Provinces 108 doctors alone died of it. The native races have proved particularly susceptible. Coming nearer home, in Spain it was "truly awful," the death-rate in Barcelona from this cause being 1,200 daily. What it has done in this country we know. How many nurses have perished in the attempt to stem its course has not yet been reckoned, but it will not surprise us if it prove to exceed considerably the number who gave their lives during the whole war.

The prevention of influenza has been shown to be quite practicable. The use of a gauze mask, such as is commonly now enforced on the nurses in fever hospitals, proved very efficacious in one of the London hospitals, where every case of influenza was isolated. Although over 1,000 cases, some very virulent, were admitted, no in-patient contracted the disease; very few of the nurses who wore their masks while in the isolation wards took the disease, and there were no deaths. Had this precaution been adopted in all hospitals there can be little doubt that the serious mortality among nurses would have been considerably reduced. Nurses at private cases, who are often exposed for many continuous hours to the infection in but partially ventilated rooms, ought to be urged by their superintendents to make use of this safeguard. The additional sense of security imparted to the mind by the protection of those organs which are known to be most pervious to infection acts in itself as a prophylactic by dispelling nervousness.

The training of V.A.D. members for future service as health visitors and welfare workers will be greatly stimulated by the decision of the Finance Committee of the Joint War Committee to grant a sum of money for the purpose of bestowing scholarships on selected candidates. Many women gave up promising work, and many interrupted their training for other careers, in order to enter the hospitals for the period of the war. They cannot, if they would, go back to the position in which war overtook them. It is in every way fitting that the Committee under which they served should provide the means through which they may enter a good career and at the same time continue their useful services to the community. It is hoped, too, that many of the Service members will be encouraged to take advanced courses in cooking and housecraft, with a view to filling some of the numerous teaching posts likely to be available.

The V.A.D. scholarship scheme is being extended so as to open up a large variety of careers for voluntary workers in hospitals. Among these are medicine and dentistry, pharmacy, and $x$-ray work. Teaching of the mentally defective is included, but the career of teaching, whether in higher or elementary schools, is omitted, which seems a pity. The age-limit is elastic enough—twenty to forty—and the only condition is that candidates for scholarships must have been working in a British unit prior to January 1917 and have continued at work as long as their services were required. In a limited number of cases the scholarships will cover the cost of living as well as tuition fees. It is a golden opportunity, and we trust there will be a sufficiency of good candidates by March 31, the date when the lists close.

The authorities of St. Mary's Hospital, Paddington, have decided to admit a limited number of V.A.D. nursing members who have completed satisfactorily two years' consecutive work in a Military Hospital. The certificate of training will be granted to these probationers at the end of three years instead of four. A good deal of uncertainty still exists as to the method in which the training for three years instead of for four will be carried out in the hospitals making this concession to members of the V.A.D. If it means that the probationer will be put through the mill from the beginning as though she had never entered a hospital, then she will lose the advantages and opportunities which crown the fourth year of training. If on the other hand she starts on her career as a second year probationer (and this is of course what she would like to do), she loses the exact drilling for the lack of which no subsequent experience can make up. Opinion in some quarters is inclining to the belief that at any rate half the first year's routine ought to be spared the V.A.D. nurse, but special organisation will be needed to provide for her missing nothing vitally necessary for her complete equipment as a nurse.

At the meeting of the Claygate Village Nursing Association, at which Lady Foley presided, it was announced that the nurse's salary had been raised to £10 a month in view of the extra cost of living. Lady Foley expressed grateful appreciation of the self-sacrifice and loyalty which Nurse Buckley had shown in remaining at her post with the Association during the war, though she might, with her skill and experience, have gone to work in a very interesting field. It is satisfactory to find that the invaluable, though inconspicuous, services rendered to the community by the nurses who stayed at home are being recognised and rewarded.

**ROUND THE HOSPITALS**—(*continued*).

The Tilam and Codishead urban districts, near Manchester, which are rapidly growing into populous centres, are already taking action under the Maternity and Child Welfare Act. The committee appointed to deal with the question recommend that the administrative arrangements be kept in the hands of the District Councils, that salaried midwives (one in each ward) be appointed, and a full-time nurse engaged who should combine home nursing with health visiting and instruction in hygiene. It is also recommended that in-patient accommodation be provided for lying-in mothers, and that a convalescent home be provided on the Derbyshire hills for nursing mothers and children under five.

---

The first decisions of the Penal Board of the new Central Midwives Board for Scotland are highly characteristic of the class of offences which the institution of this body is intended to end. One was a case of still-birth unreported; in another the midwife failed to send for medical assistance in the case of a child suffering from serious skin eruptions and broke other rules which roused doubts as to her ability to take pulse and temperature; the third was a bad case of ophthalmia neonatorum unreported and the midwife's name was struck off the roll. The gradual elimination of the unfit midwife is a most necessary if difficult part of the measures designed for the protection of infant life and it is very satisfactory that this process has now been begun in Scotland. We note that Sir John Lorne Macleod has been appointed Representative of the Convention of Royal Burghs in place of the late Sir Robert Kirk Inches.

The new Industrial Fatigue Research Board has been constituted in order to make inquiries into the effect of fatigue on output and on workers. Members of the Board are looking into such matters as the proper length of spells of work, the best way to introduce rest pauses, meal-times and night shifts. They invite suggestions which may be sent to the headquarters of the Board, 15 Great George Street, Westminster. It is earnestly to be hoped that the Board will give due consideration in the course of their researches to the case of hospital and private nurses. Very much might be done to lessen the fatigues of nursing in addition to the obvious remedy of shortening the hours. Matrons who have experimented in this direction would do well to communicate with the Board. To carry out researches merely in regard to the workers who are commonly called industrial would be to lose a great opportunity.

---

Two notorious cases have brought to public notice the spread of the drug habit among men and women alike. Legislation has not yet said its last word, and some new restrictions will probably be placed on the importation and sale of narcotics and other "remedies" for sleeplessness. But the real need is to teach people the awful danger to which they expose themselves when they try these short cuts to soothe ever-stimulated nerves. There can be no reasonable doubt that the strain and excitement of the war has led to over-stimulation. Women in particular drink more wine and resort oftener to artificial forms of speeding up their energy than they used to do. Every Ladies' Club has discovered this. It is a danger which none have better opportunities at their disposal of countering than nurses. But are nurses always alive to the fatal weakness which impels some natures to drugs as the moth to the candle? It is quite certain that many doctors are strangely oblivious of the dangers arising out of the prescription of "sedatives." The habit more often than not is formed under medical advice and continued because, the patient's will-power being sapped, he cannot keep his feet from the easy path to destruction once it has been shown him.

---

It is good to realise that the British through the Joint Committee of the Red Cross and St. John were able to render substantial succour to the Italians in the deadly straits to which they were exposed by the Austrian advance and retreat. We have already recounted the dreadful desertion by the Austrians of ravaged hospitals destitute of everything except the untended sick, the dying, and the dead, who lay in the most abject filth. Now we learn through the Duchess of Aosta, who has been retailing the facts in a letter to the British Ambassador at Rome, that wherever help was needed there the British Red Cross Commissioner, Sir Courtauld Thomson, was to be found, with cars full of every kind of food, medicines, etc., to help the starving people on either side of the line. A depôt was established at Trieste, and convoys of lorries were sent there and to other places in Austria with food and medical supplies for Allied prisoners, mostly in hospital. A travelling Red Cross kitchen dispatched into Austria to meet and feed the starving prisoners straggling back on foot towards Italy was an immense comfort, and saved many valuable lives.

---

The Red Cross pearls have done very well indeed. From first to last they have brought in close on £100,000 to the Red Cross, and the finest necklace fetched £23,000. So much virtue lies in a mere idea.

